# A distinct population of Low-Density Granulocytes with unique features associated with subclinical vascular alterations in systemic lupus erythematosus

**DOI:** 10.3389/fimmu.2026.1794775

**Published:** 2026-05-21

**Authors:** Uxía Tobío-Parada, Javier Rodríguez-Carrio, Aleida Martínez-Zapico, Ángel I. Pérez-Álvarez, Silvia Suárez-Díaz, Ana Suárez, Patricia López

**Affiliations:** 1 Group of Basic and Translational Research in Inflammatory Diseases. Department of Functional Biology, Immunology Area, Faculty of Medicine, University of Oviedo, Oviedo, Spain; 2 Instituto de Investigación Sanitaria del Principado de Asturias (ISPA), Oviedo, Spain; 3 Department of Internal Medicine, Hospital Universitario Central de Asturias, Oviedo, Spain; 4 Department of Neurology, Hospital Universitario Central de Asturias, Oviedo, Spain

**Keywords:** antigen presenting cells (APC), cardiovascular disease (CVD), flow cytometry, low-density granulocytes (LDGs), systemic lupus erythematosus (SLE)

## Abstract

**Background:**

Low-density granulocytes (LDGs) play a key role in the pathogenesis of Systemic Lupus Erythematosus (SLE) and has been associated with vascular complications. Their heightened activation and NETosis may contribute to immune dysregulation and vascular inflammation. In this study, we analyzed a distinct myeloid subset excluded from standard LDG/monocyte gating due to high CD14 and CD15 co-expression.

**Methods:**

Myeloid cell populations were quantified by flow cytometry in freshly isolated blood mononuclear cells (PBMC) from SLE patients (n=143), non-disease controls (n=42) and non-autoimmune atherosclerotic individuals (n=22). High-dimensional clustering and visualization of myeloid cells were performed using the FlowSOM algorithm. Circulating levels of cellular subsets were analyzed in relation to subclinical carotid arteriopathy determined by ultrasonography and/or previously documented clinical cardiovascular (CV)-disease.

**Results:**

High-dimensional analysis identified a distinct CD14^+^CD15^+^CD16^+^ population in PBMC from patients and controls, separate from known LDG/monocytes. Phenotyping in 15 SLE patients characterized them as CD66b^+^CD15^+^ neutrophils displaying a CD14^+^CD16^+^HLADR^+^CD10^low^ profile, thus naming them APC-like neutrophils (nAPC-like). nAPC-like correlated with Th1 (ρ=0.230; p=0.006), Th17 (ρ=0.244; p=0.004) and Treg (ρ=-0.219; p=0.010) cells in SLE, as well as with SLEDAI and anti-dsDNA titers (all p<0.001). Moreover, nAPC-like were increased in patients and controls with traditional CV-risk factors or clinical/subclinical CV-disease. A minor CD16^dim_^nAPC-like subset (CD10^neg^CD66b^high^CD15^low^), suggestive of an immature status, was expanded in SLE and correlated with serum IL-6 and BLyS (p<0.05).

**Conclusions:**

Our findings reveal a previously unrecognized LDG subset with neutrophil and APC-like features that may contribute to immune dysregulation and subclinical carotid arteriopathy in SLE and non-autoimmune individuals.

## Graphical Abstract

**Figure:**
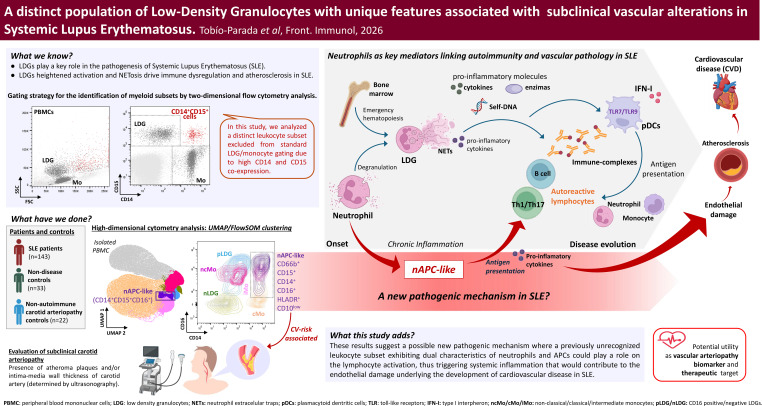

## 1 Introduction

Neutrophils are critically involved in the pathogenesis of Systemic Lupus Erythematosus (SLE) and its associated cardiovascular complications, exhibiting a complex and multifaceted role. Under the chronic inflammatory conditions typically present in SLE, neutrophil subpopulations undergo phenotypic and functional alterations that perpetuate tissue damage and disease progression. Notably, a distinct subset of neutrophils known as low-density granulocytes (LDGs) has been identified in the peripheral blood of SLE patients, representing a heterogeneous population with potentially diverse origins. LDGs may arise in peripheral tissues due to aberrant activation processes and dysregulated granulopoiesis of neutrophils. Alternatively, certain immature LDGs may be prematurely mobilized from the bone marrow in response to systemic inflammation (1, 2). These LDGs are characterized by an enhanced capacity to release neutrophil extracellular traps (NETs), which are enriched in oxidized mitochondrial DNA. The release of these NETs triggers the activation of other immune cells, including plasmacytoid dendritic cells (pDCs), subsequently driving the overproduction production of Type-I interferon (3), a key immunological hallmark of SLE. Moreover, NET components can function as autoantigens, directly stimulating auto-reactive lymphocytes and promoting the production of pathogenic autoantibodies and immune-complexes. Enzymes derived from neutrophils, such as myeloperoxidase, further contribute to endothelial dysfunction, vascular injury, and atherosclerotic plaque development (3). In this line, previous own studies have revealed an increased presence of total circulating LDGs, with a notable expansion of those lacking CD16 expression (nLDG), which was associated with vascular abnormalities in patients with SLE and chronic kidney disease (1, 2).

In this study, we identified a distinct subset of CD15^+^CD14^+^HLA-DR^+^ low-density neutrophils with variable CD16 and CD10 expression, exhibiting an antigen-presenting cell (APC)-like phenotype in SLE and healthy donors. We further evaluated their relationship with immune dysregulation and subclinical carotid arteriopathy, thus suggesting their potential utility as early biomarkers of vascular alterations and as targets for preventive therapeutic strategies.

## 2 Patients and methods

### 2.1 Patients and controls

One-hundred and forty-three SLE patients fulfilling the American College of Rheumatology (ACR) classification criteria (4) were sequentially recruited from the outpatient clinic of the Autoimmune Disease Unit (Hospital Universitario Central de Asturias, HUCA). Clinical manifestations and cardiovascular (CV) events during the disease course were obtained after a retrospective review of clinical records whereas anti-dsDNA titer and SLE disease-activity-index (SLEDAI) were calculated at sampling. Also, treatments received over the previous 3 months were recorded (**Table 1**). Additionally, thirty-three sex- and age-matched volunteers from the same geographic area without any pathology or treatment were enrolled as non-disease controls. Furthermore, a separate group of 22 individuals with carotid arteriopathy, determined by ultrasonography and without any autoimmune condition, was recruited from the Department of Neurology (HUCA) to participate as a disease control group, allowing comparison with individuals presenting classical atherosclerotic disease.

**Table 1:** Demographic and clinical features of SLE patients and control groups.

	SLE patients (n=143)	Non-disease controls (n=33)	Non-autoimune arteriopathy controls (n=22)
Demographic features			
Sex, n (female/male subjects)	130/13	31/2	4/18***
Age, years (mean ± SD)	47.23 ± 13.14	45.36 ± 11.07	77.86 ± 6.01***
Serum biochemical parameters, (mean ± SD)			
Total cholesterol, mg/dl (mean ± SD)	184.40 ± 34.39	189.12 ± 32.37	147.95 ± 30.04***
HDL cholesterol, mg/dl (mean ± SD)	60.97 ± 18.07	63.93 ± 13.81	43.65 ± 13.20***
LDL cholesterol, mg/dl (mean ± SD)	105.39 ± 29.89	109.00 ± 30.71	77.20 ± 24.85***
Triglycerides, mg/dl (mean ± SD)	94.62 ± 50.96	79.42 ± 31.16	149.40 ± 69.98***
Traditional CV risk factors, n (%)			
Dyslipidemia	26 (18.18)	3 (9.10)	13 (59.10)***
Hypertension	34 (23.77)*	2 (6.06)	20 (90.90)***
Diabetes (type II)	3 (2.09)	0 (0.00)	12 (54.55)***
Obesity (BMI>30)	20 (13.99)	1 (3.03)	7 (31.82)**
Smoking habit	33 (23.07)	4 (12.12)	0 (0.00)**
**Subclinical arteriopathy, n (%)**	46 (32.17)*	5 (15.15)	22 (100)***
**CV disease, n (%)**	17^a^ (11.89)***	0 (0.00)	22^b^ (100)***
SLE clinical manifestations			
Age at diagnosis, years (mean ± SD)	35.09 ± 14.45		
Disease duration, years (mean ± SD)	12.58 ± 11.62		
SLEDAI score (mean ± SD)	3.47 ± 4.22		
Anti-dsDNA/titer, U/ml (mean ± SD)	107 (83.39 ± 222.86) .03 ± 226.25		
Treatment, n (%)			
None or NSAIDs	6 (4.20)		
Antimalarial drugs	126 (88.11)		
Glucocorticoids	60 (41.96)		
Immunosuppressive drugs^c^	43 (30.07)		

BMI, body mass index; dsDNA, double stranded DNA; RF, rheumatoid factor; NSAID, nonsteroidal anti-inflammatory drug.

^a^CV complications of SLE patients consisted of cerebrovascular disease (n=4), heart disease (n=9) and peripheral vascular disease (n=5).

^b^CV disease of non-autoimmune arteriopathy patients: ischemic cardiopathy (n=7); peripheral vascular disease (n=4); lacunar stroke (n=5); cardioembolic stroke (n=5); contralateral atherothrombotic stroke (n=3).

^c^Mycophenolate mophetil, azathioprine.

Differences between disease subgroups and control subgroups for each parameter were analyzed by χ^2^ test, T tests or U-Mann Whitney test as appropriated (*p<0.05, **p<0.01, ***p<0.001).

Fresh blood samples from all participants were tested for cell count and serum lipid analyses (total cholesterol, high- and low-density lipoprotein cholesterol and triglycerides). Information on traditional cardiovascular risk factors (hypertension, dyslipidemia, diabetes, smoking, and obesity) was obtained for all participants. The occurrence of subclinical arteriopathy was assessed by Doppler ultrasound at the Department of Neurology (HUCA) based on the presence of carotid plaques and/or an intima-media wall thickness of the internal carotid artery (cIMT) >0.9 mm, following the Mannheim consensus (5). A plaque was defined as a distinct area protruding into the vessel lumen of at least 0.5 mm, with 50% greater thickness than the surrounding cIMT or the presence of cIMT>1.5 mm.

Participants were classified into study groups according to diagnosis and cardiovascular status: (i) non-disease controls without tCVR factors, (ii) non-disease controls with tCVR, (iii) SLE patients without tCVR, (iv) SLE patients with tCVR, (v) SLE patients with clinical or subclinical vascular disease, and (vi) non-autoimmune individuals with carotid arteriopathy. This classification enabled the evaluation of associations between circulating nAPC-like cell levels and vascular involvement within each diagnostic category, as well as exploratory comparisons between autoimmune and non-autoimmune vascular disease.

The study was approved by the Regional Ethics Committee for Clinical Research (Servicio de Salud del Principado de Asturias) according to the Declaration of Helsinki. All individuals signed a written informed consent prior to participation in the study.

### 2.2 Flow cytometry

Monocytes, low-density granulocytes (LDGs) and T-cell lymphocytes were quantified by flow cytometry in SLE, healthy controls and non-autoimmune atherosclerotic individuals. Monocyte subpopulations (classical, intermediate, and non-classical), as well as LDGs, were quantified in freshly peripheral blood mononuclear cells (PBMCs) isolated by density gradient centrifugation and stained with anti-CD14 (Immunostep), anti-CD15 (Miltenyi Biotec), anti-CD16 (Immunostep), as previously described (1). Total monocytes were defined as CD14^+^CD15^neg/low^ and total LDG as CD14^neg/low^CD15^+^ cells within the PBMC gate. Then, classical (CD14^++^CD16^-^), intermediate (CD14^++^CD16^+^) and non-classical (CD14^+^CD16^+^) subsets were discriminated against total monocytes, whereas the expression of CD16 and CD14 in LDG allowed the discrimination of CD16^neg/low^CD14^neg^ (nLDG) and CD16^pos^CD14^low^subsets. Additionally, a detailed phenotypic characterization was performed in a subgroup of 15 SLE patients (CD14, CD15, CD16, CD10, HLA-DR, CD66b).

The percentage of Treg (Foxp3^+^CD127^-^CD25^high^), Th1 (IFNγ^+^) and Th17 (IL-17^+^) cells were calculated in reference to the amount of gated CD4^+^ lymphocytes present in fresh blood samples. Firstly, cells were extracellularly stained with monoclonal antibodies specific to CD4, CD25 (Immunostep) and anti-CD127 (eBioscience). Then, cells were fixed, permeabilized (“Fixation/permeabilization buffer set”; eBioscience) and intracellularly stained with anti-Foxp3 and IFNγ or IL-17A (eBioscience), following the manufacturer’s instructions.

For all analyzed populations, cells stained with the corresponding isotype-matched irrelevant antibodies were used as negative controls (eBioscence). Data acquisition was performed on a BD FACSCanto II flow cytometer. The analysis was based on cells located in a plot-area termed “the living region” which was defined using forward and side-scatter and using FlowJo™ v10 software (BD). Results were expressed as the percentage of positive cells.

### 2.3 High-dimensional analysis of myeloid populations

Myeloid cells were gated, and 15,000 events per sample were randomly selected using the DownSample plugin and subsequently concatenated. The concatenated file was used for dimensionality reduction using the uniform manifold approximation and projection (UMAP) algorithm. To further explore and identify cell populations, FlowSOM clustering was applied. Finally, marker expression levels across clusters were visualized using the ClusterExplorer plugin. All analyses were performed within FlowJo™ v10 software (BD).

### 2.4 Cytokine quantification

Serum levels of IL-6 and BLyS were quantified by LEGENDplex (BioLegend) by using a FACS Canto II flow cytometer (BD), following the manufacturer’s instructions. The lower limits of detection were (pg/ml) 1.10 for IL-6 and 10.40 for BLyS.

### 2.5 Statistical analysis

All the data are presented as medians (interquartile ranges, IQR) unless otherwise stated. The Kolmogorov-Smirnov test was used to assess the normality of the distribution of the data. Statistical differences among groups were assessed using the χ² test for categorical variables and the Mann–Whitney U test or Kruskal–Wallis test for continuous variables, as appropriate. When applicable, p-values were adjusted for multiple comparisons using the two-stage linear step-up procedure of Benjamini, Krieger, and Yekutieli to control the false discovery rate (reported as FDR-adjusted q-value). Correlations between variables were performed by using Spearman’s rank correlation test, with multiple testing corrected applied using the Benjamini-Hochberg (BH) method; only statistically significant correlations after adjustment (adjusted p-values, *p*adj) are reported. To account for potential confounding by demographic variables, multivariable linear regression models including age, sex and traditional cardiovascular risk factors as covariates were performed to determine the association between circulating nAPC-like or CD16^dim^-nAPC-like cells and vascular involvement in SLE and control subgroups. To this end, variables were log-transformed to achieve normal distribution and standardized linear regression coefficients (β) were used as an estimate of the association. A p-value<0.05 was considered to indicate statistical significance. The data were analyzed using R-Studio software version 4.3.1 (R-Core Team).

## 3 Results

### 3.1 Phenotypic characterization of APC-like neutrophils: a new CD14^+^CD15^+^ subset with APC markers

**Figure 1:**
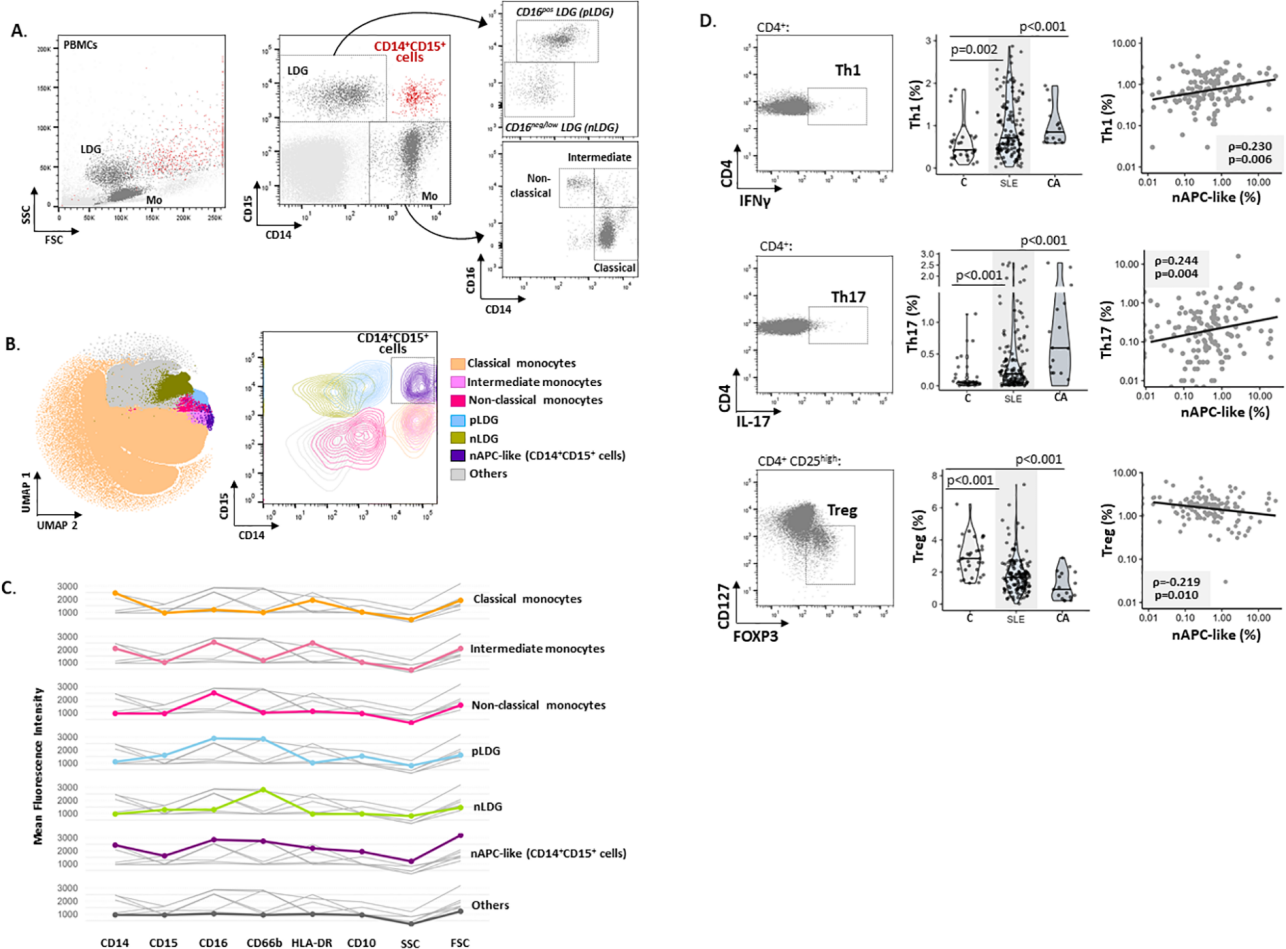
Analysis and characterization of a novel PBMC-CD14^+^CD15^+^ subset. **(A)** Gating strategy to identify monocytes and low-density granulocyte (LDG) subsets from controls and patient groups by flow cytometry. Representative dot-plots from a SLE patient showing FSC (forward side scatter) *vs* SSC (size side scatter) and CD14 *vs* CD15 gating used for conventional identification of LDGs and monocytes (Mo). **(B)** UMAP analysis of myeloid cells using FlowSOM clustering (left) and contour-plot showing with CD14 and CD16 expression (right). **(C)** Marker expression profile plots were generated using MFI (mean fluorescence intensity) values for CD14, CD15, CD16, CD66b, HLA-DR, CD10, SCC and FSC channels. These values represent the average marker expression across each population identified via UMAP and FlowSOM clustering. Color lines represent the expression profile of each individual cellular subset identified in the FlowSOM clustering in comparison with the rest of cellular populations (grey lines). **(D)** Correlation between nAPC-like and T-cell subsets in SLE patients. Dot-plots from a representative patient show the gate strategy for the identification of Th1, Th17 and Treg cells in fresh blood samples. Violin graphs display median frequency values of T-cell subsets (percentage *vs* CD4^+^ cells) in all the analyzed groups. Scatter-plots show frequency values of T-cell *vs* nAPC-like subsets (percentage *vs* myeloid cells). Statistical differences were evaluated using the Mann–Whitney U-test and correlation analyses by Spearman tests.

The analysis of myeloid cells commonly present in the isolated PBMC fraction from SLE patients and controls by conventional two-dimensional flow cytometry revealed to us the presence of a minor cellular subset which was excluded from our own standard gating strategies for the identification of LDGs and monocytes (1) due to their simultaneous expression of the highest CD14 and CD15 levels (**Figure 1A**). Then, high-dimensional clustering and visualization of PBMC-myeloid cells from patients and controls using the FlowSOM algorithm confirmed the presence of a distinct CD14^+^CD15^+^CD16^+^ population, separate from the two LDGs subsets previously reported (CD16^neg/low^ and CD16^pos^-LDG, nLDG and pLDG, respectively) (1) as well as the three canonical monocyte subsets (classical, intermediate and non-classical monocytes) (**Figure 1B**).

Further phenotypic characterization in a small group of SLE patients (n=15) identified these cells as neutrophils (CD66b^+^CD15^+^) higher in size and granularity than conventional LDGs and expressing HLA-DR, CD14, CD16 and low levels of CD10 (**Figure 1C**). Therefore, since they share features of neutrophils and antigen presenting cells, we named them APC-like neutrophils (nAPC-like). According to this suggested ability to activate T-lymphocytes, the nAPC-like population displayed positive correlations with Th1 (CD4^+^IFN^+^) and Th17 (CD4^+^IL-17^+^) cells, both subsets increased in SLE compared to controls. Moreover, Treg cells were downregulated in SLE and exhibited a negative association with the nAPC-like subset (**Figure 1D**).

### 3.2 Association of nAPC-like with subclinical vascular arteriopathy and inflammation

**Figure 2:**
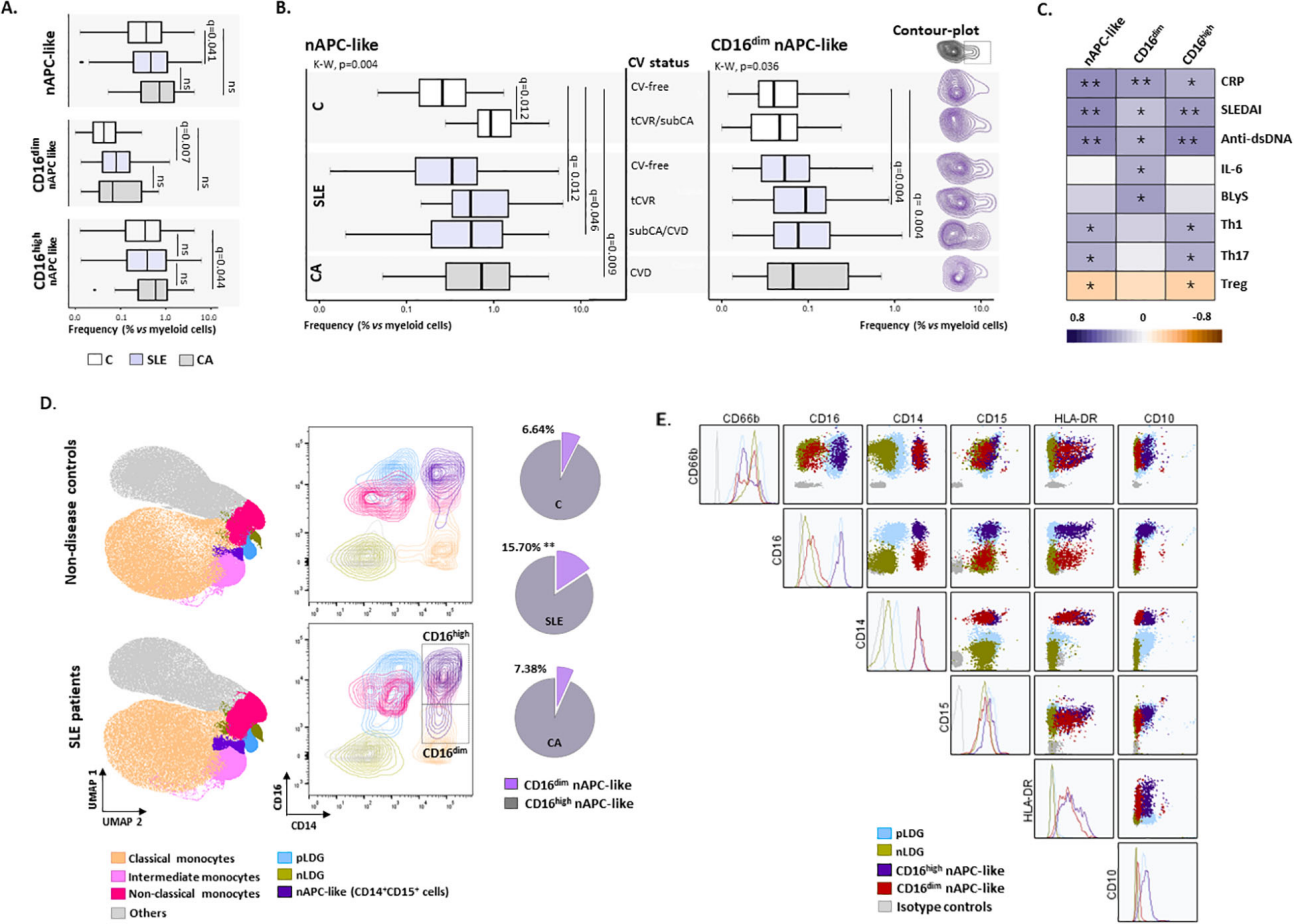
nAPC-like populations in relation to cardiovascular disease (CVD). **(A)** Frequency of nAPC-like and their CD16^dim/high^ subpopulations in SLE patients and control groups. Box-plots show the frequency of nAPC-like relative to myeloid cells in non-disease controls **(C)**, SLE patients and non-autoimmune carotid arteriopathy controls (CA). **(B)** nAPC-like population (left panel) and CD16^dim^ nAPC-like subset (right panel) in relation to CVD affectation. Box-plots show the frequency of nAPC-like relative to myeloid cells in SLE patients and group controls stratified according to the presence of absence of traditional risk factors (tCVR and CV-free, respectively), subclinical arteriopathy (sub-AP) or cardiovascular disease (CVD) at enrolment. The line within each box represents the median value. Statistical differences among each individual group and healthy CV-free controls were evaluated by the Kruskal–Wallis test (K-W p-value). Further statistical analyses across all groups displayed in each plot were conducted with correction for multiple comparisons using the two-stage linear step-up procedure of Benjamini, Krieger, and Yekutieli to control the false discovery rate. All pairwise comparisons across groups were statistically tested with FDR correction. Only statistically significant pairwise comparisons are displayed in the figure for clarity (FDR-adjusted q-values); complete results of all comparisons (including non-significant ones) are provided in **Supplementary Table 1**. **(C)** Correlation matrices among nAPC-like subsets and clinical parameters in SLE patients, where the color of the tiles is proportional to the strength of the correlation between each pair of variables. Correlations were assessed using Spearman tests with multiple testing correction applied using the Benjamini-Hochberg (BH) method (adjusted p-values are shown: *padj<0.05, **padj<0.01). **(D)** UMAP plots and contour-plot showing distribution of different myeloid subsets according to CD14 and CD16 expression from healthy donors (up) and SLE patients (down). Pie charts show the median proportion of CD16^dim^ and CD16^high^ nAPC-like subsets in relation to the total amount nAPC-like cells in controls and SLE groups. (Mann–Whitney U-test, SLE *vs* HC, **p<0.01). **(E)** Overly histograms and dot-plots show the distribution of CD16^dim^ nAPC-like, CD16^high^ nAPC-like, nLDG and pLDG subsets based on the expression of CD66b, CD14, CD15, HLA-DR, CD10, CD16 markers, as well as their SSC and FSC characteristics.

The frequency of circulating nAPC-like cells was evaluated across non-disease controls, SLE patients, and individuals with non-autoimmune carotid arteriopathy (**Figure 2A**). Although no significant differences were observed in the nAPC-like cell population between SLE patients and controls, relevant qualitative differences emerged when considering cardiovascular status. In controls, nAPC-like were elevated in individuals with tCVR or subclinical arteriopathy (n=8) compared to their CV-free counterparts (**Figure 2B**) and positively correlated with carotid intima-media thickness (cIMT) (ρ=0.492, *p*adj=0.013) and C-reactive protein (CRP) levels (ρ=0.368, *p*adj=0.042). Similarly, these cells were increased (**Figure 2B**) and correlated with cIMT (ρ=0.531, *p*adj=0.026) in non-autoimmune arteriopathy patients, thus supporting an association of nAPC-like with vascular endothelial alterations. In SLE patients, nAPC-like were significantly increased in patients with tCVR or clinical/subclinical vascular involvement. Moreover, they were correlated with CRP, SLEDAI and anti-dsDNA titters (**Figure 2C**). Furthermore, in a multivariable regression analysis restricted to the SLE cohort and including age, sex and tCVR factors (hypertension, dyslipidemia, diabetes, smoking and obesity) as covariates, total circulating nAPC-like cells remained independently associated with vascular involvement (β=0.192; p=0.045). No significant associations were observed between circulating nAPC-like cells and other clinical, immunological or therapeutic related variables.

Notably, it is worth noting that the nAPC-like population includes a minor CD16^dim^ nAPC-like subset (**Figure 2D**), barely detected in controls and non-autoimmune arteriopathy patients, but significantly expanded in SLE patients, particularly in those displaying tCVR factors, subclinical carotid arteriopathy or CVD (**Figure 2B**). This increase remained significant in SLE patients in a multivariable model including sex, age, tCVR factors and presence of carotid plaque across all individuals (β=0.178, p=0.038). In contrast, the CD16^high^ nAPC-like subset was more prominently increased in individuals with carotid arteriopathy (**Figure 2A**). These findings highlight that disease-associated changes in nAPC-like cells are driven by specific subsets, with the CD16^dim^ population emerging as the main contributor in SLE.

Additional multivariable linear regression analysis adjusted by sex and age confirmed that vascular involvement remained significantly associated with increased circulating nAPC-like cell levels in controls with tCVR factors or subclinical arteriopathy (β=0.477; p=0.007), as well as in SLE patients with tCVR (β=0.315; p=0.013) and clinical/subclinical vascular disease (β=0.293; p=0.021). Similarly, higher levels of CD16^dim^-nAPC like cells were independently associated with tCVR (β=0.340; p=0.040) and vascular involvement (β=0.281; p=0.024) in SLE patients.

The upregulation observed in the non-autoimmune carotid arteriopathy group did not remain statistically significant after adjustment for age and sex, likely reflecting the predominance of older male individuals in this group compared with non-disease CV-free controls.

Further phenotypic characterization of the CD16^dim^-nAPC-like subset showed a CD10^neg^CD66b^high^ profile with lower CD15 expression than the CD16^high^ counterparts (**Figure 2E**), suggestive of a more immature neutrophil status. Remarkably, these findings highlight the overlap between CD16^dim^ and CD16^high^ nAPC-like subsets with nLDG and pLDGs regarding CD10 and CD16 levels, thus appearing to be APC-like nLDG and APC-like pLDG, respectively.

Finally, the CD16^dim^ nAPC-like subset in SLE patients was correlated with serum IL-6 and BLyS levels, as previously reported for nLDGs (1). However, the associations previously observed with Th1, Th17 and Treg cells (**Figure 1D**) were mainly driven by the CD16^high^ nAPC-like subset), supporting a potential role in shaping T cell responses.

## 4 Discussion

Neutrophils have traditionally been considered terminally differentiated, short-lived effector cells of the innate immune system (6). Although they have long regarded as a homogeneous population, recent studies have highlighted their phenotypic and functional plasticity, including the capacity to acquire antigen presentation ability (6, 7). In this context, the present work arises from the sudden discovery of a minor leukocyte subset, migrating within the low-density fraction of peripheral blood leukocytes, characterized by a high expression of CD14 and CD15 and with a larger size than monocytes and LDGs. These cells were present in the circulation of SLE patients and healthy controls associated with subclinical vascular arteriopathy risk and raised C-reactive protein, a classical inflammatory marker, whereas in SLE patients were also related to disease activity (SLEDAI and anti-dsDNA titer). Further phenotypic analysis revealed that these cells exhibit a neutrophil-like surface profile (CD66b^+^CD15^+^) while also expressing markers of antigen presenting cells (APC), such as MHC class II (HLA-DR), thus referred to here as APC-like neutrophils (nAPC-like).

A growing body of evidence suggests that neutrophils may exhibit distinct phenotypes and perform diverse functions depending on the tissue microenvironment and the specific disease context (6, 8). Several studies demonstrate that neutrophils upregulate APC-associated surface molecules such as CD11c, MHC-II and T-cell co-stimulatory molecules when cultured with cytokines (e.g., GM-CSF) or autologous T-cells, while retaining core neutrophil phenotypic and functional features (6). In fact, neutrophils with antigen-presenting capabilities have been previously described in various pathological settings, including cancer, infections or autoimmune diseases (6, 9, 10). For instance, in patients with rheumatoid arthritis, neutrophils in the synovial fluid express large amounts of MHC-II molecules and then stimulate T-cell proliferation (10). In SLE patients, Mysore et al. reported that blood neutrophils expressing APC markers—including CD11c, CD80 and HLA-DR—were elevated and positively correlated with SLEDAI (11), suggesting that they may contribute to lupus pathogenesis. Moreover, they also showed that sera from SLE patients —or immune complexes formed *in vitro* using IgG isolated from lupus patients and ribonucleoproteins— were able to induce the conversion of polymorphonuclear blood neutrophils into APC-like cells with mononuclear-like appearance (11). It remains to be determined whether these cells overlap with or are distinct from low-density granulocytes (LDGs), which warrants further investigation.

The typical scenario in SLE is characterized by a chronic and systemic inflammatory environment that promotes innate immune responses, leading to vascular endothelial activation and the subsequent infiltration of neutrophils, T cells, and other leukocytes into the vessel wall. In this context, our findings demonstrate an increase of this nAPC-like population in controls and lupus patients at CV risk, suggesting that this minor neutrophil subset may represent a circulating biomarker with carotid atherosclerotic alterations, not only in SLE patients but potentially also in the general population. Consistent with this hypothesis, individuals with non-autoimmune arteriopathy, included in the study as positive controls for CVD, also exhibited increased circulating levels of the nAPC-like population that were associated with carotid intima-media thickness (cIMT). These results reinforce the link between neutrophils with APC features and vascular alterations, in agreement with previous studies in atherosclerotic mouse models and hyperlipidemic patients, which reported increased levels of circulating neutrophils expressing antigen-presenting markers such as MHC-II, CD80 and CD86, along with evidence of functional antigen-presenting activity (12).

Neutrophil infiltration into the vascular wall contributes to the progression of atherosclerosis through the release of NETs and granule proteins such as matrix metalloproteinases (MMPs), myeloperoxidase (MPO) and elastase (3). Nevertheless, the presence of neutrophils with APC features could add a new mechanism of vascular damage. APCs uptake and present antigens to T-cells, triggering the adaptive immune response (6, 7). Thus, nAPC-like could gain the ability to present antigens and activate T-cells, resulting in the production of inflammatory cytokines, and ultimately, potentially influence vascular inflammatory processes. Along with this suggested ability to activate T-lymphocytes, the amount of nAPC-like was associated with an expansion of Th1 and Th17 cells in our SLE cohort, and with a concomitant reduction in regulatory T-cell (Treg) levels. These findings align with previous own studies linking Th1 and Th17 responses to CVD development in autoimmune conditions (1). Consistent with our observations, Zhao et al. demonstrate how the exposition to oxidized-LDL of *in vitro* activated neutrophils can induce an APC-like profile (12). This phenotype promotes atherosclerosis progression by enhancing effector Th1 responses, as evidenced by a positive correlation between neutrophils and IFN-γ–producing T-cells in both atherosclerotic mice and hyperlipidemic patients. These APC-like neutrophils also secrete elevated levels of immunostimulatory cytokines, such as IL-1β and IL-23, which are known to facilitate Th17 cell differentiation (11).

Finally, a minor subset of CD16^dim^CD10^neg^ cells were identified into the nAPC-like population, which was significantly expanded in our SLE patients but especially in those with CV-risk. This finding parallels the previously described differentiation of nLDGs and pLDGs based on CD16 expression in our earlier studies (1) based on the CD16 expression and other specific features supporting that pLDG are likely derived from mature circulating neutrophils whereas the minor nLDG subset originates from emergency granulopoiesis in inflammatory environments. Indeed, the increased expression of HLA-DR and CD14 observed in both CD16^dim^ and CD16^high^ nAPC-like subsets compared to nLDG and pLDGs, lead us to hypothesize that nAPC-like could be derived from the activation of both LDGs subsets. Of note, CD14 could be synthesized by neutrophils, and the CD14/TLR4 signaling pathway has been involved in thromboinflammation (13). Supporting this theory, Rice et al. have described the existence of hyperactive CD10^neg^ neutrophils in peripheral blood of severe COVID-19 patients (9). Similar to nLDGs, the CD16^dim^ nAPC-like subset were simultaneously CD66b^pos/high^ and CD10^neg^, a profile suggestive of an immature state (14). Taken together, our results pointed to an expansion of peripheral immature CD10^neg^CD16^neg/low^ LDGs in circulation of lupus patients at CV risk. Hence, CD10^neg^ neutrophils have been associated with an elevated susceptibility to the degranulation and production of endothelial permeability in SLE (15). In our lupus patients, the frequency of the CD16^dim^ nAPC-like, as formerly observed with the nLDG population, positively correlates with IL-6 levels, a cytokine well-known for promoting premature neutrophil release from the bone marrow through enhanced granulocyte recruitment and activation. In this line, circulating immature neutrophils have been detected in multiple diseases mediated by immune response, including cancer or COVID-19 (6). Although the CD16^dim^ subset distinguishes SLE patients from controls and reflects an inflammation-driven immature neutrophil pathway, it represents only a small fraction of total nAPC-like cells. In contrast, the more abundant CD16^high^ subset expresses higher HLA-DR levels and likely has greater antigen-presenting capacity, explaining why T-cell correlations are mainly driven by CD16^high^ cells, while CD16^dim^ subset mainly reflects disease-specific inflammatory processes linked to emergency granulopoiesis.

In summary, our results suggest a potential new pathogenic mechanism where a previously unrecognized leukocyte subset exhibiting dual characteristics of neutrophils and APCs could play a role on lymphocyte activation, thus triggering adaptive immune responses and systemic inflammation. These processes could be linked to endothelial dysfunction and vascular alterations associated with subclinical carotid arteriopathy in SLE, but also in general population, although the association with vascular involvement may partly reflect the influence of age and traditional cardiovascular risk factors. The observed association of these nAPC-like with immune dysregulation and markers of carotid vascular damage suggests that they may represent a cellular feature linked to vascular involvement in SLE and a promising therapeutic target to prevent CV comorbidities. However, further studies, particularly longitudinal and mechanistic analyses, are required to clarify the origin and functional role of nAPC-like subsets and to determine their potential clinical relevance in the vascular complications associated with SLE.

## Data Availability

The deidentified patient data underlying the main variables are not available but could be shared on reasonable request to the corresponding author. Requests to access these datasets should be directed to Patricia López, lopezpatricia@uniovi.es.
