# Supplementary material for: A distinct population of Low-Density Granulocytes with unique features associated with subclinical vascular alterations in systemic lupus erythematosus
Source: Front Immunol. 2026 May 21;17:1794775. doi: 10.3389/fimmu.2026.1794775 (PMC13233468; doi:10.3389/fimmu.2026.1794775)
Supplement: Supplementary file 1 [file Table1.docx]

**Supplementary Table S1. Complete pairwise comparisons between study groups shown in Figure 2B.**

| **Pairwise comparisons** | **nAPC-like** | | **CD16^dim^ nAPC-like** | |
| --- | --- | --- | --- | --- |
|  | **FDR-adjusted q-values** | **p-value** | **FDR-adjusted q-values** | **p-value** |
| **C-CVfree *vs* C-tCVR/subCA** | **0.012** | **0.003** | 0.635 | 0.605 |
| **C-CVfree *vs* SLE-CVfree** | 0.138 | 0.083 | 0.289 | 0.127 |
| **C-CVfree *vs* SLE-tCVR** | **0.012** | **0.002** | **0.030** | **0.004** |
| **C-CVfree *vs* SLE-subCA/CVD** | **0.046** | **0.015** | **0.030** | **0.004** |
| **C-CVfree *vs* CA** | **0.009** | **0.001** | 0.256 | 0.057 |
| **C-tCVR/subCA *vs* SLE- CVfree** | 0.095 | 0.038 | 0.651 | 0.667 |
| **C-tCVR/subCA *vs* SLE-tCVR** | 0.287 | 0.263 | 0.303 | 0.178 |
| **C-tCVR/subCA *vs* SLE- subCA/CVD** | 0.138 | 0.096 | 0.303 | 0.178 |
| **C-tCVR/subCA *vs* CA** | 0.287 | 0.273 | 0.574 | 0.403 |
| **SLE-CVfree *vs* SLE-tCVR** | 0.138 | 0.099 | 0.256 | 0.094 |
| **SLE-CVfree *vs* SLE- subCA/CVD** | 0.366 | 0.407 | 0.256 | 0.092 |
| **SLE-CVfree *vs* CA** | 0.101 | 0.048 | 0.574 | 0.477 |
| **SLE-tCVR *vs* SLE- subCA/CVD** | 0.348 | 0.360 | 0.910 | 0.999 |
| **SLE-tCVR *vs* CA** | 0.765 | 0.911 | 0.574 | 0.505 |
| **SLE- subCA/CVD *vs* CA** | 0.287 | 0.246 | 0.574 | 0.505 |

Pairwise comparisons were derived from a Kruskal–Wallis test followed by correction for multiple comparisons using the two-stage linear step-up procedure of Benjamini, Krieger and Yekutieli to control the false discovery rate. Both raw p-values and FDR-adjusted q-values are provided.
